# Health Is Wealth: Study on Consumer Preferences and the Willingness to Pay for Ecological Agricultural Product Traceability Technology: Evidence from Jiangxi Province China

**DOI:** 10.3390/ijerph182211761

**Published:** 2021-11-09

**Authors:** Ximing Chen, Jie Shang, Muhammad Zada, Shagufta Zada, Xueqiang Ji, Heesup Han, Antonio Ariza-Montes, Jesús Ramírez-Sobrino

**Affiliations:** 1 School of Economics and Management, Northeast Forestry University, Harbin 150040, China; chenximing0313@126.com (X.C.); 13576274061@163.com (X.J.); 2 Business School, Henan University, Kaifeng 475000, China; mzada@henu.edu.cn; 3 Department of Management Sciences, Alhamd Islamic University, Islamabad 45400, Pakistan; 4 Ideological and Political Education Department, School of Marxism, Northeast Forestry University, Harbin 150040, China; shaguftanefu@yahoo.com; 5 College of Hospitality and Tourism Management, Sejong University, Seoul 05006, Korea; 6 Social Matters Research Group, Universidad Loyola Andalucía, C/Escritor Castilla Aguayo, 4, 14004 Córdoba, Spain; ariza@uloyola.es; 7 Business Growth Challenges Research Group, Universidad Loyola Andalucía, C/Escritor Castilla Aguayo, 4, 14004 Córdoba, Spain; jramirez@uloyola.es

**Keywords:** ecological agricultural products, traceability technology, food safety, selective experiment method

## Abstract

The application of traceability technology is an important way to solve food safety problems. Different traceability technologies bring different effects to consumers. Existing studies have not explored consumers’ preferences in regards to product traceability technology applications, and they have not analyzed their willingness to pay. Therefore, this study focused on organic rice, an ecological agricultural product. The study was based on a survey from Jiangxi Province, China. It used a selective experiment method in order to analyze consumer preferences and the willingness to pay for ecological agricultural product traceability technology. The results show that consumer preferences are as follows: blockchain technology application attributes, traditional traceability-technology-application attributes, high credit-supervision attributes, and international-certification attributes. In terms of willingness to pay, consumers have the highest willingness to pay for the application of blockchain technology, which they are willing to pay CNY 21.902 more per kg for this attribute. At the same time, consumers are also willing to make additional payments for traditional traceability-technology-application attributes, high credit-supervision attributes, and international-certification attributes. Their willingness to pay is CNY 20.426, CNY 17.115 yuan, and CNY 11.049, respectively.

## 1. Introduction

In modern society, people pay more and more attention to food safety issues. There are laws, regulations, and industry standards in order to maintain food safety, but food safety issues continue to arise, which endanger the health of consumers [1,2]. By tracking and certifying the information of the entire food supply chain, the source of food contamination can be effectively identified and dealt with, which thereby manages safety issues in the agricultural food sector [3,4]. Traceability plays a vital role in the field of agricultural food safety. The application of traceability technology in the agricultural food supply chain can help companies improve food safety and quality, and reduce the possibility of introducing unsafe food into the market. By applying traceability technology that is coupled with specific food certification, which includes local food, organic food, and genetically modified food, the safety of agricultural food can be ensured, which also ensures that consumers understand the product’s characteristics [5].

The traditional Internet of Things (IoT) traceability system uses radio frequencies to monitor and store specific information at various stages of production, processing, distribution, and consumption, which thereby provides valuable information for agricultural food quality inspections and traceability [6]. However, this system is based on a centralized server client paradigm, and stakeholders and consumers must rely on an information point to store, transmit, and share traceability information [7]. The product information may be lost or tampered with when there is a problem with the information point. At the same time, it is difficult to establish an effective trust mechanism between the participants in the traceability chain by relying on existing traceability technology as noted by Helo and Hao and Khan and Salah [8,9], which will seriously affect cooperation between supply-chain entities.

Blockchain is the key technology of Bitcoin [10]. It is essentially a decentralized ledger that keeps transaction records on multiple computers at the same time [11]. Blockchain has some important features, such as decentralization, security transparency, unforgeability, smart contract, and verifiability [12,13,14,15]. These features can effectively solve the problem of trust and security between people [16]. The technical characteristics of blockchain allow it to have a broad application prospect in agricultural food traceability. Existing studies have found that the ability of blockchain in product traceability, authenticity, and execution of real-time transactions will greatly improve food traceability, which will have a positive impact on food quality, safety, and sustainability [17,18,19].

According to the above research, both the traditional traceability system and the latest blockchain technology have improved the access of consumers to agricultural food information to a certain extent, which is conducive in order to protect the rights and the interests of consumers. However, the application of traceability technology will increase the additional costs of the main body of the agricultural food supply chain. In order to ensure the profit level, the final cost will be shared by consumers and agricultural food supply-chain entities through an increase in the product price. Existing studies also show that consumers are willing to pay a premium for the traceability of products [20,21].

The research on consumers’ willingness to pay for traceability has accumulated a lot of achievements [22,23,24,25]. However, most of the existing studies focus on traceable food itself, less on the application of traceability technology, and less on the analysis of consumers’ willingness to pay for the application of traceability technology. The application of different traceability technologies will bring different effects to consumers. What food traceability technology do consumers prefer in order to protect their information acquisition? What is the willingness of consumers to pay for different traceability technologies? The research on these problems has important reference value for the technology application and the product price setting of agricultural food enterprises.

In order to explore consumers’ preference and willingness to pay for traceability technology in food consumption, this study presents the application preference and willingness to pay for traceability technology for ecological agricultural products in China. There are two reasons for choosing China as the research location that include: (1) China is an important agricultural country in the world, but due to food safety issues, such as melamine, its reputation in the food industry has been seriously affected, and China has vigorously promoted the application of traceability technology in the food field in recent years. (2) Blockchain has important value in the traceability application of agricultural food as a new traceability technology. The Chinese government attaches great importance to the development of blockchain and actively promotes the application of blockchain in agriculture. Some domestic consumers have a certain understanding of blockchain, and the country has launched an attempt to apply blockchain to agricultural traceability systems. The application of traceability technology for ecological agricultural products was selected for research, because ecological agricultural products have higher quality requirements than general agricultural products, and they also have stricter requirements for product traceability. The application of traceability technology to ecological agricultural products has greater value.

Compared with the existing research, this study had two extensions that include: (1) an expansion of the research perspective, where most of the past research is limited to the payment premium brought by the traceability of the product, whereas this article focuses more on consumers’ willingness to pay for traceability technology, and; (2) the expansion of research frontiers. Most of the existing research on the traceability of the agricultural food supply chain is limited to the application of traditional traceability technology. This study further incorporated the new traceability technology of blockchain into the analysis system.

Scholars focused on consumer preferences for traceable food and willingness to pay for traceable food during earlier periods [20,23,26,27,28,29,30,31,32,33,34,35,36]. With the continuous deepening of research, scholars not only pay attention to the traceable food itself, but also further explore other attributes of the product. Based on the utility theory by Lancaster (1966), it is not the product itself but the attributes of the product that satisfy the consumer’s utility. According to the existing research, scholars are concerned about the traceability information attributes of traceable food [23,31,32,37,38,39,40,41,42,43,44,45,46,47,48,49,50], which have all been discussed. The technical attribute of food is also an important attribute of food. The application of different traceability technologies will affect the amount of traceable food information consumers can obtain and the authenticity of the information. However, the existing research has not yet discussed the attributes of consumers’ application of traceability technology to food. Therefore, this study investigated Chinese consumers’ preference and willingness to pay for the traceability technology of eco-agricultural products, in order to analyze what kind of traceability technology consumers prefer to use in food, as well as to explore consumers’ willingness to pay for the security attribute of traceability technology application.

## 2. Materials and Methods

### 2.1. Selection of Test Method

The choice-test model method (CE) is a non-market evaluation method for public goods that was proposed by Louviere and Hensher (1982). Its theory is based on the consumer theory and the random utility theory. This method has been used by many scholars in order to study consumers’ willingness to pay for product attributes [49,51].

When applying the selection experiment method to estimate the selection probability of related attributes, there are three commonly used methods, which include the standard multivariate logit model (MNL), the conditional logit model (CL), and the random parameter logit model (RPL). The standard multivariate logit model among them has strict hypothesis restrictions, which must comply with the IIA hypothesis test, (IIA is irrelevant choice independence hypothesis), and it requires higher related data. The random parameter logit model is more complicated. While considering the attribute variables that change with the plan, the personal characteristics of the interviewee are also further considered, and it discusses the heterogeneity of the selection behavior. Furthermore, this research mainly discusses consumers’ preference and willingness to pay for the traceability technology of ecological agricultural products. Considering the applicability and simplicity of the model, this study applied the conditional logit model as the probability estimation model.

Assuming that the random utility function of the respondent is U(X),

(1) $$U_{ni}\left(X_{ni}\right)=V_{ni}\left(X_{ni}\right)+\varepsilon_{ni}$$

In the above formula, $U_{ni}$ is the utility function for the respondent n to choose option i from the given selection set C, $V_{ni}$ is the indirect utility function of option i selected by n, $X_{ni}$ is the attribute feature of scheme i selected by n, and $\varepsilon_{ni}$ is the random interference item of n selection scheme i. (Random interference items represent random factors that cannot be controlled or easily measured. It includes that the investigator only knows the content of a single option or is forced to make choices).

According to the principle of maximum utility, the probability of n choosing i from the selection set C is

(2) $$P\left(U_{i}\right)=P⎣\left(V_{ni}+\varepsilon_{ni}\right)>\left(V_{nj}+\varepsilon_{nj}\right)⎦;\;i\neq j,\;\;\;i,j\in C$$

Assuming that ε obeys the extreme value distribution, the probability of n choosing i from the selection set *C* can be expressed by the Logit model as

(3) $$P\left(U_{i}\right)=\frac{\exp(\mu V_{ni})}{\sum_{K\in C}\exp(\mu V_{ni})}$$

Its linear form can be expressed as

(4) $$V_{ni}=C_{i}+\sum_{j}\beta_{j}X_{ij}$$

In the above formula, $C_{i}$ represents the substitution of a specific constant. $\beta_{j}$ represents the coefficient of the j-th attribute $X_{ij}$ of the i-th scheme. Based on this, the marginal value of the willingness to pay for each attribute can be expressed as

(5) $$WTP=-\left(\beta_{attribute}\right)/\beta_{M}$$

In the above formula, $\beta_{attribute}$ is the estimated coefficient of each attribute item, $\beta_{M}$ is the marginal utility of the average payment, which is usually expressed by the estimated coefficient of the payment item.

### 2.2. Property Selection

The product selected in the experiment of this study was organic rice, because rice is the most important food item for Chinese people, and organic rice is an important part of China’s ecological agricultural products. According to the selection experiment method, this article believes that organic rice has the following four attributes, which include traceability technology application attributes, credit-supervision attributes, certification-type attributes, and price attributes. Three levels were set for each attribute, which are shown in Table 1.

Traceability-technology-application attributes and level settings: the application of traceability technology is an important technical attribute of ecological agricultural products. The traceability-technology-application attribute contains three levels. The first level is no traceability technology, which is the reference standard. This mean that organic rice does not use any traceability technology, and it is difficult for consumers to obtain traceability information. The second level is the application of traditional traceability technology, such as the IoT traceability system that was mentioned above. The application of traditional traceability technology can guarantee consumers’ access to product traceability information to a certain extent, but it is relatively limited. The third level is the application of blockchain. The application of blockchain in product traceability can greatly improve the authenticity of traceable information and protect consumers’ access to traceable information.

Setting the attributes and levels of credit supervision: credit supervision is an effective means to ensure the application of traceability technology. Through the implementation of credit supervision, the actual application of traceability technology in the ecological agricultural product supply chain is restricted, and it is ensured that the main body of the supply chain uses the traceability technology instead of false propaganda. The attribute of credit supervision can be divided into three levels that include no credit supervision, which is reference standards, low-level credit supervision, which is government-led credit supervision, and high-level credit supervision, which is a diversified credit supervision that is constructed by multiple entities, such as the government and third-party regulatory agencies.

Setting the authentication attributes and levels: the certification of product conditions is another guarantee for the application of its traceability technology. Certification has an important impact on consumers’ purchasing behavior (Yu et al., 2014; Gracia and de-Magistris, 2016; Lusk et al., 2018). This study divided the certification attributes into three levels that include no certification, which is the reference standard, national certification, and international certification.

Setting the price attributes and levels: offsetting the price attributes was key in order to carry out the selection experiments. The price attributes can be used to calculate consumers’ willingness to pay for each attribute of traceable products. Based on the survey of domestic organic rice prices in China, where the average sale price of organic rice in China in 2018 was CNY 23.56/kg, this study comprehensively considered the convenience of the research and set the basic price at CNY 25/kg. According to the principle of isometric setting, the price attributes were divided into three levels, which included CNY 25/kg, CNY 30/kg, and CNY 35/kg.

According to the attribute setting and the hierarchical division of this study, 81 (3 × 3 × 3 × 3) different solutions could be produced. In order to improve the efficiency of the investigation and ensure the resolution of the investigation, this study conducted an orthogonal design of the plan, and obtained nine independent and irrelevant plans composed of different attribute-state levels. By taking into consideration the convenience of the survey respondents in order to conduct the survey, and the reality of the plan, which was combined with the attribute variables, nine kinds of plans were combined to form four selection sets after eliminating combinations that were impossible to exist in reality. Each selection set included three program options that included one basic program option, which was composed of reference standards for various attributes, and two improvement program options. One selection set is shown in Table 2 below.

### 2.3. Selection of Survey Samples

For this survey, we used Jiangxi Province, China, as the main survey site. Jiangxi is an important agricultural province in China. Furthermore, rice is the staple food in Jiangxi Province. The local residents have a high demand for rice. In recent years, the provincial government has vigorously advocated the concept of green environmental protection. Therefore, the local residents have a higher demand for organic rice and other ecological agricultural products. In the survey, we randomly selected Nanchang, Jiujiang, Ji’an, and Ganzhou in Jiangxi Province in order to conduct the research, and each city issued 100 questionnaires. In addition to the selection set, the questionnaire also included basic information, such as gender, age, education level, and income.

## 3. Results and Discussion

### 3.1. Demographic Characteristics

Table 3 includes the descriptive statistics of the basic status of the 353 valid survey respondents, which include gender, age, education, monthly income, and region of the surveyed consumers. Most of the surveyed consumers were male, accounting for 59.77%, and females accounted for 40.23%. The surveyed consumers’ ages mainly ranged between 19 and 45, which accounted for 82.44% of the total. The monthly income of the surveyed consumers mainly ranged between CNY 3000 and 6000, which accounted for 56.66% of the total. Ji’an had the most effective response rate of the questionnaires among them, accounting for 27.20% of the total.

### 3.2. Regression Results of the Conditional Logit Model

The conditional logit estimation was conducted using Stata software, and the model was tested. The results are shown in Table 4 below. It can be seen from Table 4 that LR chi2(7) = 137.950 and Prob > chi2 = 0.00, and this reflects that the model’s likelihood ratio test passed, which indicates that the model is effective. In addition, Pseudo R2 = 0.179, which indicates that the selected attribute variables have a high degree of explanation in regards to the consumer choice behavior, and the attribute selection is more appropriate.

It can be seen from Table 4 that the traditional-traceability-technology-application coef. (TT) is 1.246, which is significantly positive at the 1% statistical level. The blockchain technology application coef. (BT) is 1.336, which is significantly positive at the 1% statistical level. This shows that both the application of the traditional traceability technology and the application of the blockchain technology can have a positive impact on consumer utility, and the application of blockchain technology has a greater positive impact on consumer utility than the application of traditional traceability technology. This also shows that consumers are more inclined to buy ecological agricultural products using blockchain technology than traditional traceability technologies. This is because blockchain technology not only guarantees more information that is obtained than the traditional traceability technology, but it also depends on the distributed technology in order to ensure the authenticity and security of the product information, so consumers can obtain more, and more authentic, product information.

The low credit supervision coef. (DS) was 0.070, but it was not statistically significant. This shows that a certain degree of credit supervision can have a positive impact on consumer utility, but the low-level credit supervision constructed by the government alone is not reliable, which is due to the lack of independent participation of third parties and other independent institutions. Furthermore, it is difficult to obtain the full trust of consumers. This is due to regulatory organizations that are only composed of government departments lacking external constraints, which are prone to bribery and other illegal activities in the process of practice, so it is difficult to effectively supervise the application of traceability technology. Therefore, consumers’ preference for this attribute is not significant.

The high credit-supervision coef. (HS) was 1.044, which was significantly positive at the 1% statistical level. It shows that consumers prefer high-level credit supervision the most in terms of the credit-supervision attributes, and it also shows that a diversified supervision system that involves third-party independent agencies and governments can gain the full trust of consumers. This was due to the interweaving of various forces under the higher credit supervision, which can guarantee the reasonable application of traceability technology to a higher extent, so consumers can obtain relevant information through traceability technology in order to protect their own rights and interests.

The national certification coef. (GC) was not significant, and the international’s coef. (IC) was 0.674, which is significant at the statistical level of 1%. It reflected that Chinese consumers prefer international certification with product certification, and the ecological agricultural products with international certification are more recognized by consumers. This was because in the current practice, China’s domestic ecological agricultural product certification system is chaotic, and various certification rules are more complicated. Furthermore, there are certain private transactions in the certification process, and the certification results are recognized differently by consumers.

In order to further ensure the reliability of the model, this study further used the hybrid logit model to conduct a robustness test. The results are shown in Table 5. The independent variable and dependent variable settings of the mixed logit model are consistent with the conditional logit model. In addition, this article further incorporates the interviewee’s personal characteristics variables into the hybrid logit model. It can be seen from Table 5 that the coefficients of the attribute variables estimated by the mixed logit model are similar to the conditional logit model. Among them, the variables TT, BT, HS, IC, and PR maintain the same direction in the conditional logit model estimation and the mixed logit model estimation, and they all have high significance. This shows that the result of conditional logit model estimation is relatively robust. In terms of AIC and BIC values, the conditional logit model is slightly lower than the mixed logit model. Therefore, the conditional logit model has a higher goodness of fit.

### 3.3. Consumer Willingness to Pay

In order to understand consumers’ willingness to pay for product attributes, such as traceability technology application attributes, the *WTP* needed to be further calculated. The *WTP* represents the amount of currency that consumers are willing to pay for this attribute in order to keep the utility unchanged when a certain attribute of the traceable product changes. According to the estimated parameter values in Table 4, the average willingness to pay for each attribute of the ecological agricultural products is calculated using Equation (5). The specific calculation results are shown in Table 6.

The data in Table 6 show that consumers have a positive willingness to pay for the traceability-technology-application attributes and guarantee attributes. Consumers have the highest willingness to pay for blockchain technology application attributes among them. Compared with the non-technical application attributes, consumers’ willingness to pay for this attribute is CNY 21.902. Secondly, consumers are also willing to pay for the application of traditional traceability technology. Compared with non-technical application attributes, consumers’ willingness to pay for the application of traditional traceability technology is CNY 20.426. In the calculation of the willingness to pay for the traceability-technology-application guarantee attribute, the willingness to pay for the higher credit-supervision attribute (HS) is CNY 17.115, and the international-certification attribute (IC) is CNY 11.049.

## 4. Conclusions and Countermeasures

In this study, the choice-test method was used to explore consumers’ preference regarding the application attributes and the guarantee attributes of ecological agricultural product traceability technology through the conditional logit model. Results show that, without considering the personal characteristics of consumers, consumers’ preferences for the application attributes and security attributes of the traceability technology of ecological agricultural products are as follows: technology-application attributes, traditional-traceability-technology-application attributes, high credit-supervision attributes that are jointly constructed by the government and third-party independent regulatory agencies, and international-certification attributes. Secondly, in terms of willingness to pay, consumers have the highest willingness to pay for blockchain technology application attributes, and consumers are willing to pay an additional CNY 21.902 per kg for this attribute. At the same time, consumers are willing to pay extra for the application attribute of traditional traceability technology, the advanced credit-supervision attribute, and the international-certification attribute, which their willingness to pay is CNY 20.426, CNY 17.115, and CNY 11.049, respectively.

Combined with the above conclusions, this study puts forward the following countermeasures and suggestions.

1. Accelerate the application of blockchain technology in the agricultural supply chain. The application of blockchain can improve the traceability of the agricultural food supply chain and can protect the rights and interests of consumers. At the same time, consumers also have a higher willingness to pay. For this reason, the agricultural food supply chain needs to accelerate the application of blockchain. On the one hand, it is necessary to strengthen the construction of the blockchain infrastructure in the supply chain, further update the blockchain hardware facilities on the basis of the existing traceability system of the Internet of Things, and accelerate the application of blockchain technology. On the other hand, it is necessary to strengthen the knowledge update of the supply-chain members, conduct special training about the blockchain application for the relevant technical personnel, and improve the ability of the supply-chain members in order to apply blockchain.
2. Strengthen the construction of the traceable information credit-supervision system. Credit supervision is an important foundation in order to ensure the exact application of traceability technology. In order to promote the organic combination of credit supervision and traceability technology, it is necessary on the one hand to speed up the construction of diversified regulatory agencies, which includes the main body of the supply chain, third-party independent institutions, and the government, to supervise the application of traceability technology and the real situation of relevant information in order to improve the independence of supervision. On the other hand, it is necessary to speed up the construction of the network credit information query platform in a timely manner and publish the list of dishonesty, so there is no place for dishonest people to hide.
3. Learn from international standards in order to improve the certification system of ecological agricultural products. China’s domestic eco-agricultural product certification system is mixed, and the certification results lack consumer trust, which is not conducive to ensuring that the application of product traceability technology after certification is trusted by consumers. As a result, China needs to learn from the mature eco-agricultural product certification system of the international community, simplify the certification process of the eco-agricultural products, improve the transparency of the certification process information, and enhance consumer trust.

The existing research has not yet discussed consumers’ application of traceability technology attributes to food. This study used Chinese consumers’ preference and willingness to pay for the application of traceability technology for ecological agricultural products, in order to analyze consumer’s application of traceability technology attributes to food. The preference and willingness to pay for its protection attributes are an extension of this article. However, due to the limitation of data samples and space, there is no further discussion about the impact of consumers’ personal characteristics on their attribute selection, and there are no further analyses on the heterogeneity of consumers’ attribute selection for traceability technology applications, which will be improved in the next stage of research. In the next stage, the research group will expand the sample for a more in-depth analysis.

## 5. Future Outlook

Existing research has not discussed the traceability technology attribute of food application by consumers, but this study investigated the application preference and willingness to pay of Chinese consumers for the traceability technology of ecological agricultural products, so as to analyze consumers’ preference and willingness to pay for the traceability technology attribute of food application and its guarantee attribute, which is the expansion of this paper. As for the fitting model used in this paper, although the conditional logit model relaxes the IIA hypothesis test to analyze the subject’s preference for a variety of options, it does not fully consider the possible impact of the subject’s own heterogeneity on its preference. Therefore, in the next stage, the research group will conduct in-depth analysis on the impact of consumer heterogeneity on the application preference of traceability technology for ecological agricultural products. In terms of methods, more-advanced methods such as a random parameter logit model will also be adopted to make up for the shortcomings of the conditional logit model.

## Figures and Tables

**Table 1:** Traceable organic rice attributes and their level settings.

Attributes	Level	Symbol	Variable Assignment
Traceability technology application attributes	No application of traceability technology	NT	Yes = 1; No = 0
	Apply traditional traceability technology	TT	Yes = 1; No = 0
	Application of blockchain	BT	Yes = 1; No = 0
Credit-supervision attributes	No credit supervision	NS	Yes = 1; No = 0
	Low credit supervision	DS	Yes = 1; No = 0
	Higher Credit Supervision	HS	Yes = 1; No = 0
Certification-type attributes	No certification	NC	Yes = 1; No = 0
	National certification	GC	Yes = 1; No = 0
	International Certification	IC	Yes = 1; No = 0
Price attributes	CNY 25/kg	Price	25
	CNY 30/kg		30
	CNY 35/kg		35

**Table 2:** Selection set example.

	Option A	Option B	Option C (Reference)
Traceability technology application	Traditional traceability technology	Blockchain technology	No traceability technology
Credit supervision	Higher credit supervision	Low credit supervision	No credit supervision
Brand	No certification	No certification	No certification
Price	CNY 30/kg	CNY 35/kg	CNY 25/kg
Select			

**Table 3:** Distribution of the characteristics of the survey samples.

Feature	Personal Characteristics	Frequency	Relative Frequency
Gender	Male	211	59.77%
	Female	142	40.23%
Age	Under 18 years old (including 18 years old)	26	7.37%
	19–30 years old (including 30 years old)	130	36.83%
	31–45 years old (including 45 years old)	161	45.61%
	45–60 years old (including 60 years old)	33	9.35%
	Over 60 years old	2	0.57%
Education	Primary school and below	12	3.40%
	Junior middle school	85	24.08%
	Senior high school and technical Secondary school	165	46.74%
	Junior college	55	15.58%
	Bachelor degree or above	35	9.92%
Monthly Income	CNY 3000 and below	17	4.82%
	CNY 3000–6000 (including CNY 6000)	200	56.66%
	CNY 6000–9000 (including CNY 9000)	69	19.55%
	CNY 9000–12,000 (including CNY 12,000)	52	14.73%
	Over CNY 12,000	14	3.97%
Region	Nanchang	78	22.10%
	Ji’an	96	27.20%
	Jiujiang	94	26.63%
	Ganzhou	84	23.80%

**Table 4:** Conditional logit estimation.

y	Coef.	Std. Err.	z	*p* > |z|	95% Conf.	Interval
TT	1.246	0.238	5.240	0.000	0.780	1.712
BT	1.336	0.259	5.150	0.000	0.828	1.845
DS	0.070	0.246	0.280	0.776	−0.412	0.552
HS	1.044	0.245	4.260	0.000	0.564	1.524
GC	−0.297	0.285	−1.040	0.297	−0.855	0.261
IC	0.674	0.348	1.940	0.053	−0.008	1.355
PRICE	0.061	0.020	2.970	0.003	0.021	0.101
LRchi2(7)	137.950					
Prob > chi2	0.000					
Log likelihood	−316.230					
Pseudo R2	0.179					
AIC	646.46					
BIC	681.17					

**Table 5:** Estimation results of mixed logit.

y	Coef.	Std. Err.	z	*p* > |z|	95% Conf.	Interval
TT	1.970	0.895	2.200	0.028	0.215	3.724
BT	1.145	0.477	2.400	0.016	0.210	2.081
DS	−0.096	0.306	−0.310	0.753	−0.695	0.503
HS	1.083	0.328	3.300	0.001	0.440	1.726
GC	0.526	0.542	0.970	0.331	−0.535	1.588
IC	1.326	0.479	2.760	0.006	0.386	2.266
PR	0.106	0.038	2.770	0.006	0.031	0.181
LRchi2(7)	86.47					
Prob > chi2	0.000					
Log likelihood	−301.624					
AIC	637.248					
BIC	702.833					

**Table 6:** Consumers’ willingness to pay for traceability technology applications and guarantee attributes (CNY/kg).

Attributes	Willingness to Pay
TT	20.426
BT	21.902
HS	17.115
IC	11.049

## Data Availability

The data presented in this study are available on request from Corresponding authors. The data are not publicly available due to privacy.
